# Exome sequence analysis and follow up genotyping implicates rare *ULK1* variants to be involved in susceptibility to schizophrenia

**DOI:** 10.1111/ahg.12226

**Published:** 2017-11-17

**Authors:** Mariam M. Al Eissa, Alessia Fiorentino, Sally I. Sharp, Niamh L. O'Brien, Kate Wolfe, Giovanni Giaroli, David Curtis, Nicholas J. Bass, Andrew McQuillin

**Affiliations:** ^1^ Molecular Psychiatry Laboratory, Division of Psychiatry University College London London UK; ^2^ Current address: Institute of Opthalmology University College London London UK; ^3^ University College London Genetics Institute University College London London UK

**Keywords:** association, olanzapine, burden analysis

## Abstract

Schizophrenia (SCZ) is a severe, highly heritable psychiatric disorder. Elucidation of the genetic architecture of the disorder will facilitate greater understanding of the altered underlying neurobiological mechanisms. The aim of this study was to identify likely aetiological variants in subjects affected with SCZ.

Exome sequence data from a SCZ cas–control sample from Sweden was analysed for likely aetiological variants using a weighted burden test. Suggestive evidence implicated the UNC‐51‐like kinase (*ULK1*) gene, and it was observed that four rare variants that were more common in the Swedish SCZ cases were also more common in UK10K SCZ cases, as compared to obesity cases. These three missense variants and one intronic variant were genotyped in the University College London cohort of 1304 SCZ cases and 1348 ethnically matched controls.

All four variants were more common in the SCZ cases than controls and combining them produced a result significant at *P* = 0.02.

The results presented here demonstrate the importance of following up exome sequencing studies using additional datasets. The roles of *ULK1* in autophagy and mTOR signalling strengthen the case that these pathways may be important in the pathophysiology of SCZ. The findings reported here await independent replication.

## 1 INTRODUCTION

Schizophrenia (SCZ) is a serious psychiatric disorder with an estimated lifetime prevalence of 1% (Merikangas et al., 2011, Shivashankar et al., 2013). The main clinical features of SCZ are hallucinations, delusions, and disorganized speech and behaviour (McGuffin et al., 2003; Cardno & Gottesman, 2000). SCZ may give rise to severe debilitating clinical manifestations that impact affected individuals, their families, and their caregivers (Nurnberger & Berrettini, 2012). Evidence for the heritability of SCZ has been provided by twin and family studies. As stated in a recent review, the estimated genetic heritability for SCZ is between 60% and 90% (Neale & Sklar, 2015).

Genome‐wide association (GWA) studies have been widely used to identify genetic risk factors of small to medium effect size in genetically complex disorders. This approach has proved successful in the study of SCZ (Neale & Sklar, 2015). However, rare single base changes of large effect size have proved more difficult to identify. A study of exome‐sequenced Swedish SCZ subjects and controls revealed an excess of rare coding variants among cases and was able to implicate particular enriched gene sets, but no single gene achieved statistical significance after correction for multiple testing (Purcell et al., 2014).

We had previously applied weighted burden analysis tests to whole exome sequencing data from these 5090 Swedish SCZ case and control subjects and to data from a UK‐based case–case sample from the UK10K project, consisting of 982 obese cases and 1392 SCZ cases (Curtis, 2015; Curtis & UK10K Consortium, 2016). This method of analysis tested for an excess of variants that had been weighted according to rarity and predicted effects on function, such that stop variants were weighted more highly than nonsynonymous (NS) changes, which were weighted more highly than synonymous variants and the like . The scores for each variant are summed within subjects and a *t*‐test is carried out to see if the total scores are higher in cases than controls. The weight of evidence implicating a gene is reported as the signed log *P*‐value (SLP), which is the base 10 logarithm of the *P*‐value given a positive sign if the excess of variants is in cases and a negative sign if the excess is in controls. Three sets of analyses were performed including either all variants, all NS variants, or all rare (MAF < 0.1) NS variants. In the analysis of the Swedish dataset, the UNC‐51‐like kinase (*ULK1*) produced SLPs of 3.1, 3.0, and 3.1 and was ranked 14 of 20,267 genes in the analysis using only rare NS variants. These gene‐wise results were largely driven by three NS variants (rs145451295, rs55815560, and rs145279005), and it was noted that an intronic variant (rs188342389) was also more common in cases than controls (Table 1). As shown in the same table, the first two of these variants were also more common in the SCZ than obese UK10K cases, although the gene‐wise results did not demonstrate a significant excess of rare, likely functional, variants in *ULK1*, with SLPs of 0.13, 0.14, and 0.25.

**Table 1:** Genotype counts and allele frequencies in the Swedish schizophrenia exome samples and the UK10K severe childhood onset obesity cases and schizophrenia cases

Variant		Swedish exomes		UK10K	
Position on chromosome 12 (hg19); predicted effect		Controls	SCZ cases	Obese cases	SCZ cases
SIFT; PolyPhen2; Mutation Taster					
rs145451295	CC	2523	2510	971	1374
132394378; T242I	CT	4	16	1	11
Tolerated; Benign; Polymorphism	MAF (%)	0.079	0.32	0.051	0.40
rs55815560	CC	2538	2533	978	1379
132401058; S665L	CT	3	9	4	10
Tolerated; Benign; Polymorphism	MAF (%)	0.059	0.18	0.20	0.36
rs145279005	CC	2535	2526	977	1384
132401539; A705V	CT	10	19	5	6
Deleterious; Probably damaging; Disease causing	MAF (%)	0.20	0.37	0.25	0.22
rs188342389	CC	2528	2523	969	1369
132405837; intronic	CT	13	20	13	22
N/A; N/A; Polymorphism	MAF (%)	0.26	0.39	0.66	0.79

The effect of the variant is shown as the amino acid change at the relevant peptide position of *ULK1* (NP_003556.1).

Taking together the gene‐wise results from the Swedish dataset and the fact that two of the variants were commoner in schizophrenia cases in both datasets, we sought to follow up these results by genotyping the variants in our own case–control sample.

## 2 MATERIALS AND METHODS

The potential aetiological impact of the three NS variants was assessed using SIFT (Sorting Tolerant From Intolerant) (Sim et al., 2012), PolyPhen‐2 (polymorphism phenotyping) (Adzhubei et al., 2010), and MutationTaster (Schwarz et al., 2014) as shown in Table 1.

We proceeded to genotype three NS and one intronic variants in the *ULK1* gene in the University College London (UCL) dataset of SCZ cases and controls that have been described previously (Fiorentino et al., 2014; O'Brien et al., 2014). Briefly, the cases and controls were unrelated individuals of white British ancestry. The 1304 SCZ cases had their diagnoses confirmed according to the Research Diagnostic Criteria (Spitzer, Endicott, & Robins, 1978) after having been interviewed with the lifetime version of the Schizophrenia and Affective Disorders Schedule (SADS‐L) (Spitzer & Endicott, 1977). The controls comprised 868 subjects recruited with an absence of personal history of mental illness as well as an absence of mental illness in first‐degree relatives. An additional 480 controls consisted of random blood donors whose cell‐line DNA was purchased from the European Collection of Authenticated Cell Cultures, Public Health England, Porton Down, UK). DNA from case and control subjects collected by us was derived from whole blood and saliva samples. DNA extraction was performed using standard techniques. DNA was quantified using fluorimetry (Qubit, ThermoFisher, Paisley, UK)

Genotyping was performed using a competitive Allele‐Specific PCR system (KASPar, LGC Genomics, Hoddesdon, UK) on a LightCycler480 real‐ time PCR machine (Roche, Burgess Hill, UK). Tests of allelic association were performed using Fisher's exact tests with the “fisher.test” commands in R (R Core Team, 2014). We also noted the frequencies of these variants in the European 1000 Genomes subjects and in the non‐Finnish European subjects from the ExAC “nonpsychiatric” cohorts (1000 Genomes Project Consortium et al., 2012, Lek et al., 2016).

## 3 RESULTS

The results for those subjects successfully genotyped are presented in Table 2, which shows that all four variants were more common in the SCZ cases than controls. No variant was individually statistically significant, but no subject carried more than one of them so the counts could be combined and overall 32 cases and 17 controls carried one of these variants, a result with one‐tailed significance of *P* = 0.02. However, it should be noted that although the allele frequencies were higher in cases than controls, for all four variants the frequency was higher still among ExAC subjects, and, with the exception of rs188342389, for the 1000 Genome subjects.

**Table 2:** Genotype counts and allele frequencies in the UCL schizophrenia case‐control sample and allele frequencies in the European subjects from 1000 Genomes project and from the non‐Finnish European subjects in the “nonpsychiatric” ExAC cohorts

Variant		*UCL* Controls	*UCL* SCZ cases	1000 Genomes	ExAC
rs145451295	CC	1243	1250	497	17,671
	CT	2	5	6	114
	MAF (%)	0.080	0.20	0.60	0.26
rs55815560	CC	1265	1270	500	19,776
	CT	5	9	3	149
	MAF (%)	0.20	0.35	0.30	0.37
rs145279005	CC	1250	1277	498	19,622
	CT	1	4	5	100
	MAF (%)	0.040	0.16	0.50	0.25
rs188342389	CC	1251	1264	498	20,390
	CT	9	14	5	246
	MAF (%)	0.36	0.55	0.50	0.60

The overall number of cases compared with controls carrying one of these variants is significant at *P* = 0.02 (one‐sided)

## 4 DISCUSSION

The results we present demonstrate a consistent effect across different samples. In the Swedish SCZ exomes we noted suggestive evidence for an increase in rare, functional variants in *ULK1* with a gene‐wise SLP of 3.1. Four variants commoner in cases were jointly observed to be more common in UK10K SCZ cases than obesity cases and in our own SCZ cases than controls. However, we note that in two reference datasets, 1000 Genomes and ExAC, the variants were commoner than in the UCL controls and in some instance commoner than the cases. This might reflect that our result is a false positive or may be an artefact of differences in ethnicity and/or genotype calling methodologies. *ULK1* was not specifically highlighted in the original analysis of the Swedish dataset nor in the larger follow‐up analysis, both of which reported results only for variants predicted to disrupt gene functioning, which would not included the variants we genotyped (Purcell et al., 2014; Genovese et al., 2016).

The *ULK1* gene codes for a 1050 amino acid serine/threonine kinase protein. There is evidence that the phosphorylation status of ULK1 mediates the protein's regulation of autophagy. Amongst the proteins and compounds that have been shown to alter phosphorylation (either directly or indirectly) of ULK1 is AMP‐activated protein kinase (AMPK), a cellular energy sensor that phosphorylates ULK1 under conditions of glucose starvation. ULK1 phosphorylation is counterbalanced by the protein's interaction with mechanistic target of rapamycin kinase (mTOR) complex 1 (mTORC1; a downstream component of the rapamycin sensitive mTOR signalling system) under conditions of nutrient sufficiency (Kim, Kundu, Viollet, & Guan, 2011). However, the mechanism by which ULK1 promotes autophagy remains unclear (Egan et al., 2015). Interestingly, it has been suggested that both autophagy and disruption of the mTOR signalling system may play a role in the pathophysiology of SCZ (Merenlender‐Wagner et al., 2013; Gururajan & van den Buuse, 2014). The antipsychotic olanzapine drug has also been shown to activate both the AMPK and the mTOR signalling pathways (Schmidt et al., 2013).

It is expected that exome sequencing studies of complex diseases will reveal rare variants, which, with current sample sizes, will fail to generate results of strong enough statistical significance to definitively implicate specific genes. However, if such variants are genotyped in additional samples, as we have done, then cumulative evidence will eventually allow the identification of those results which are true positives. Although our results are only of borderline significance, we recommend that the variants reported here should be studied in additional datasets. Only by following such an approach will it become possible to decide which genes should be investigated using functional studies.
